# COVID-19 in Smokeless Tobacco Habitués: Increased Susceptibility and Transmission

**DOI:** 10.7759/cureus.8824

**Published:** 2020-06-25

**Authors:** Ridhima B Gaunkar, Aradhana Nagarsekar, Karla M Carvalho, Praveen S Jodalli, Kennedy Mascarenhas

**Affiliations:** 1 Public Health Dentistry, Goa Dental College and Hospital, Goa, IND; 2 Prosthodontics, Goa Dental College and Hospital, Goa, IND; 3 Oral and Maxillofacial Pathology, Goa Dental College and Hospital, Goa, IND; 4 Public Health Dentistry, Yenepoya Dental College and Hospital, Mangalore, IND

**Keywords:** smokeless tobacco, covid-19, saliva, immunity

## Abstract

As the coronavirus disease (COVID-19) pandemic continues to sweep across the globe, the world is responding by implementing public awareness campaigns, social distancing measures, and other preventive strategies to arrest the spread of this lethal disease. Infection with the severe acute respiratory syndrome coronavirus 2 (SARS-CoV-2) exacts a heavy toll on patients with existing comorbidities. Smokeless tobacco (SLT) consumption is of particular concern in countries in South Asia with high population densities, as it facilitates exposure to SARS-CoV-2 within or between communities by the act of public spitting. Salivary droplets generated in this act are a potential threat because they can transmit this airborne infection. Moreover, large gatherings at tobacco retail outlets, frequent hand-to-mouth contact, and sharing of apparatus by SLT habitués could also aid in increasing the spread of SARS-CoV-2. SLT-induced higher expression of angiotensin-converting enzyme 2 receptors along with the presence of furin in the oral mucosa and dysfunctional immune responses among SLT habitués increase viral dissemination and an individual’s susceptibility to COVID-19. Issuing rigorous regulations to restrict the use of various forms of SLT products and the obnoxious act of spitting in public can assist in arresting the spread of COVID-19. Widespread education campaigns enlightening the community regarding the adverse effects of SLT consumption and its relationship with COVID-19, along with providing effective assistance to quit for those who are addicted, would decrease the spread of COVID-19.

## Introduction and background

The coronavirus disease (COVID-19) pandemic continues to sweep across the globe, with the severe acute respiratory syndrome coronavirus 2 (SARS-CoV-2) infecting over five million people and killing over a quarter million worldwide to date. A pall of uncertainty and fear has descended upon the world, and the SARS-CoV-2 virus has brought changes to all aspects of life. According to the World Health Organization (WHO), SARS-CoV-2 is transmitted through the spread of virus-containing droplets (i.e., <2-m distance) and/or contaminated surfaces [1].

The WHO announced that COVID-19 could become an endemic disease, although they warned that it is difficult to predict its course. To control the devastation caused by the infection, the world is responding with an amalgamation of strategies: social distancing, lockdowns (complete, partial, only in areas of high incidence), testing (voluntary and compulsory, focusing on risk groups), and a plethora of other prophylactic measures [1]. The biggest challenge lies in identifying and isolating asymptomatic carriers and preventing exponential community transmission [2]. Systemic disease states such as diabetes mellitus, hypertension, and cardiac and pulmonary diseases increase disease susceptibility and mortality [3].

The results from previous studies suggest that deleterious habits such as cigarette smoking and vaping could increase the susceptibility to COVID-19 [4]. There has not been much research on the increased risk of contracting COVID-19 for smokeless tobacco (SLT) users, although the use of these products is widely prevalent in South Asia and the Western Pacific region. In this review, we explore the possibility of increased susceptibility and infectivity of SLT habitués to COVID-19.

## Review

SLT is a broad group of unburned tobacco products that are used orally (chewed and spat out) or inhaled nasally. Many Asian and Western Pacific countries use an array of SLT products such as snus, tobacco tooth powders, snuff, gutkha, khaini, tobacco powder, mawa, jarda, mishri, and tobacco paste [5-7]. Currently, the South Asian region accounts for 90% of the global consumption of SLT, with a majority in Bangladesh (25%), India (22%), and Myanmar (21%) [8].

SLT is a stimulant that causes an increase in heart rate, blood pressure, and epinephrine levels. There is a strong and proven association between the use of SLT and death due to cardiovascular disease, cerebrovascular disease, and cancer [9]. The increased cellular tropism in oral mucosa and altered immune response among SLT habitués can increase an individual’s susceptibility to COVID-19 infection. This, compounded by the act of public spitting, frequent hand-to-mouth contact, and sharing of apparatus among SLT habitués, could potentially aid in increasing disease transmission.

Smokeless tobacco habitués: increased cellular tropism in the oral mucosa (tongue) for COVID-19

The recognition of viral host cell receptors and their interaction with the host cell is crucial in studying viral tissue tropism and pathogenesis. An individual’s susceptibility to viral infections is attributed to the presence of a host cell surface attachment site (receptor) and a conducive intracellular environment to favor virus replication and release [10].

Coronaviruses belong to the family of Coronaviridae and contain a large, single, plus-stranded ribonucleic acid (RNA) genome. There is a spike protein (S glycoprotein) on the surface of SARS-CoV-2 that binds to angiotensin-converting enzyme 2 (ACE2) receptors on the host cell membrane and facilitates viral entry into target cells. The S glycoprotein is primed by host membrane proteases (i.e., proprotein convertase) and then cleaved (at the S1/S2 cleavage site) by the host-derived enzyme furin into two subunits, S1 and S2. The S2 subunit facilitates viral and host membrane fusion through the heptad repeats HR1 and HR2 [11-14].

Studies have shown that the ACE2 receptor is expressed in the oral epithelial cells of the tongue, buccal mucosa, gingiva, minor salivary gland ducts, T and B lymphocytes, and fibroblasts of the oral sub-mucosa. This suggests that SARS-CoV-2 exhibits tropism for the oral cavity [15]. SARS-CoV-2-positive individuals exhibit oral symptoms such as amblygeustia and dry mouth, which could be explained by the dysfunction of the ACE2 receptors in virally infected oral tissue [2].

**Figure 1:**
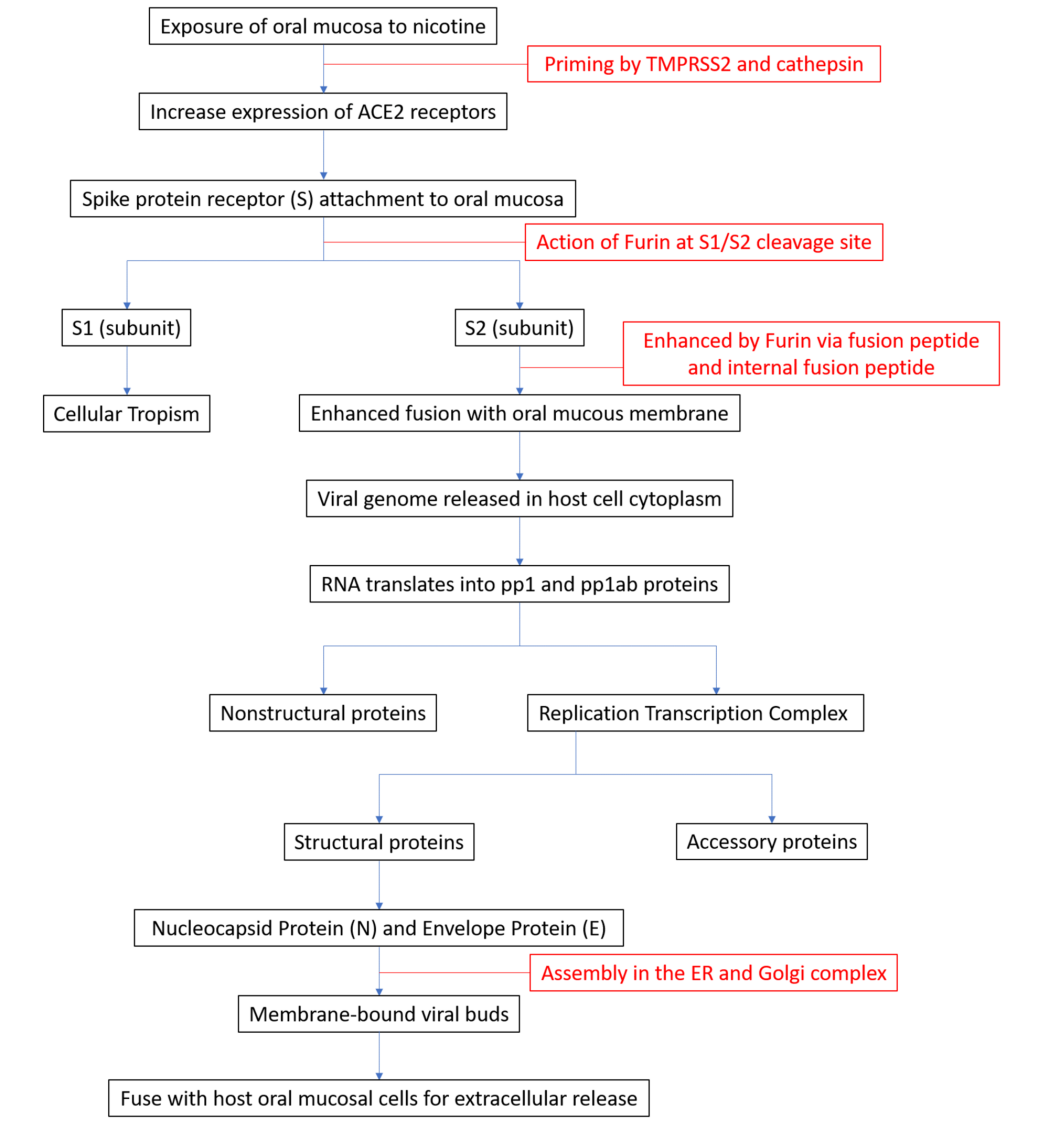
Pathogenesis: increased cellular tropism for COVID-19 in the oral mucosa and tongue of smokeless tobacco habitués The known action of the enzyme furin and the nicotine-induced increased expression of the ACE2 receptor result in COVID-19 viral tropism to the oral mucosal tissues in smokeless tobacco habitués [11-19]. TMPRSS2, transmembrane protease serine 2; ACE2, angiotensin-converting enzyme 2; ER, endoplasmic reticulum; COVID-19, coronavirus disease

Several inherent factors could result in increased infectivity of the oral cavity by SARS-CoV-2 [12]. Immunostaining studies have revealed a high expression of the furin enzyme in the human tongue [13,14]. The presence of a furin-like cleavage site in the viral spike protein enhances the invasiveness of the virus to the host cell [12]. The nicotine-induced increased expression of the ACE2 receptor in the oral mucosa would favor viral tropism [16]. Thus, SLT induces higher expression of ACE2 receptors, and, combined with the presence of furin in the oral mucosa, there would be an increased susceptibility of SLT habitués to COVID-19 (Figure 1) [11-19].

Smokeless tobacco habitués: comorbidity due to an altered immune response

It has been shown that frequent and prolonged use of various forms of SLT causes immunosuppression by affecting the adaptive (helper T cells, CD4+CD25+ regulatory T cells, CD8+ T cells, B cells, and memory T/B lymphocytes) and innate (dendritic cells, macrophages, and natural killer cells) immune mechanisms. Chronic inflammation of oral mucosa caused by SLT results in a release of prostaglandins and cytokines such as interleukin-6, interferon-α, tumor necrosis factor, and transforming growth factor β at the site of irritation, which could potentiate a pro-inflammatory cytokine response as well as cause immune system dysfunction [20-22].

When virally infected immune cells disrupt the cellular and humoral immune response, this subjects SLT habitués to cardiovascular, respiratory, and autoimmune diseases, as well as allergies, cancers, and transplant rejection [22]. It has also been shown that the group of COVID-19 patients with the worst outcomes had at least one of these comorbid conditions [3].

**Figure 2:**
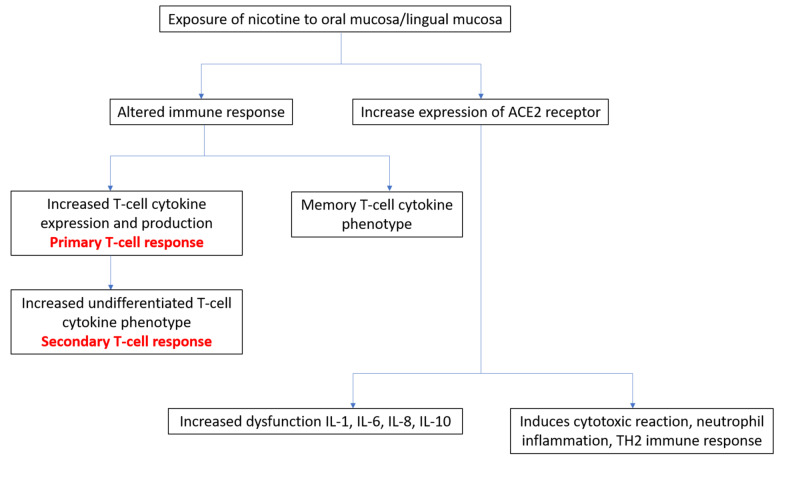
Smokeless tobacco habitués: comorbidity due to an altered immune response The nicotine-induced increased expression of ACE2 receptors on antigen presenting cells/dendritic cells of the oral mucosa as well as the T and B lymphocytes within the connective tissue result in a dysfunctional immune response mounted against the virus and in increased viral dissemination and tissue damage (through cytokine storm) in smokeless tobacco habitués. Aberrant CD4+ and CD8+ T lymphocytes result in the increased production of IFN-γ and GM-CSF. Aberrant CD14+ and CD16+ inflammatory oncocytes result in increased IL-6 production. Increased IL-8 causes increased homing of neutrophils to virally infected tissue and increased natural killer cell recruitment [20-22]. ACE2, angiotensin-converting enzyme 2; IL, interleukin; IFN, interferon; GM-CSF, granulocyte macrophage colony-stimulating factor

This dysfunctional immune response in SLT habitués against SARS-CoV-2 results in increased viral dissemination and cellular destruction via cytokine storm. This likely increases the chances that an SLT habitué would contract COVID-19 (Figure 2) [20-22].

Smokeless tobacco habitués: spitting in public and COVID-19 outbreak

SLT chewing and spitting in public is a widespread, acceptable custom in many countries of South Asia [7]. The habit of spitting in public places unleashes a stream of saliva droplets directly in the path of commuters, pedestrians, and any nearby people [23].

Physiologically, saliva is a biomixture of crevicular ﬂuid, desquamated oral epithelial cells, and microorganisms, and may contain blood, respiratory secretions, gastric acid from reﬂux, and food debris, all of which may be infectious [24]. Infected saliva generated during spitting can increase transmission of various respiratory infections through droplets spread to the mouth, nose, or eyes of individuals who are in close proximity [17].

It has been validated that saliva is a viable sample source for SARS-CoV-2 detection when compared to nasopharyngeal or oropharyngeal swabs [25,26]. Positive results were obtained from salivary samples collected from COVID-19 patients who were analyzed using reverse transcriptase-polymerase chain reaction, which is considered the gold standard for detecting viruses in respiratory secretions and blood [26]. It has been proved that saliva can harbor the virus in an active replicative state [2,25].

Interestingly, individuals in whom pharyngeal and bronchoalveolar swabs proved to be negative showed positive salivary results on the same day [27]. During hospitalization, the median viral load of the early saliva specimens among infected patients was 3.3 × 106 copies/mL (range: 9.9 × 102 to 1.2 × 108 copies/mL). Saliva specimens collected thereafter showed reduced salivary SARS-CoV-2 RNA levels after hospitalization [28]. Another striking feature noted was that patients who had otherwise tested negative through nasal swab and had cleared from all clinical symptoms still tested SARS-CoV-2 RNA-positive, suggesting that low levels of SARS-CoV-2 RNA could be excreted in saliva even after clinical recovery [29].

SLT chewing increases salivary secretion, which is followed by a very strong desire to spit [7,23]. Saliva droplets generated by spitting are formed as particles in a mixture of moisture with droplet nuclei of microorganisms. It has been observed that saliva can form into an aerosol along the air flow and become a medium for virus transmission [30,31].

The size of the saliva droplets determines the risk of viral transmission to host cells. Large droplets (diameter > 60 μm) tend to settle in the air quickly, and, therefore, the spread is limited to individuals nearest to the aerosol source. Most communicable respiratory infections are transmitted through large droplets within a short distance or by contacting contaminated surfaces. Small droplets are likely to evaporate into droplet nuclei (diameter < 10 μm) in the environment and may be transmitted over short distances (<1 m). Long-distance aerosol transmission is also determined by the length of time that the saliva droplets reside in the air (physical decay), the period during which pathogens remain viable in saliva droplets (biological decay), and the rate of acquisition of these infective droplets [30,31].

In India, SLT consumption is a habit more commonly observed in socioeconomically deprived and less literate Indian men [32]. However, in a few states of India, the SLT consumption in women is very high and is comparable to that of the male population [8]. SLT users often deface public places in many Indian cities by spitting, a practice prevalent in tiny congested low socio-economic dwellings and urban slums. The remainder of the community is subsequently at increased risk of contracting a range of communicable diseases. India is particularly vulnerable because it bears the burden of the second largest population in the world, at approximately 420 persons/km^2^ [33]. At present, Asia’s largest slum (Dharavi in Mumbai) has become an area with very high infection rates for COVID-19, where SLT consumption is a popular part of daily life [34]. Moreover, SLT habitués gather in large numbers at retail tobacco outlets, where social distancing is not practiced.

Very recently, the Government of India passed an order under the Disaster Management Act that prohibits the sales of SLT products and bans spitting in public places. However, the order was then amended, and, under Section 51 (b), the sale of SLT products is once again permitted, but the prohibition of spitting in public places was maintained [7].

Smokeless tobacco habitués: transmission of COVID-19 by hand-to-mouth contact

Frequent hand-to-mouth contact is one of the major avenues for transmission of viral infections such as COVID-19. The actual act of SLT chewing involves placing these products inside the oral cavity using fingers several times during the day [7,35]. Moreover, it has also been observed that there is a sharing of these products at workplaces [36]. Thus, SLT habitués may be more vulnerable to COVID-19 due to the possibility of transmission from frequent hand-to-mouth contact.

Recommendations

Because SLT products are often produced by numerous methods in small cottage industries and sold in unregulated markets, effective regulation is difficult. This issue is intensified by limited research and a lack of a robust evidence base. Several parties in the WHO Framework Convention on Tobacco Control consider SLT use as a regional concern limited to Southeast Asia [37]. Eradicating the use of SLT in Asia and other parts of the world would be beneficial to human health by preventing the development of chronic disease as well as the dissemination of communicable diseases.

A uniform, incremental taxation regime, the prohibition of public spitting, bans on illegal SLT export between countries, standardized and validated testing of the content of SLT products, implementation of large and clear pictorial warnings on packaging, and forbidding sales to minors should be strictly enforced to flatten the curve of the growing SLT menace [38].

Tobacco cessation should be accelerated using evidence-based cessation and prevention strategies such as varenicline, nicotine replacement therapies, and behavioral interventions, which may help SLT users to quit [9]. There should be increased public awareness through increased social media usage during this pandemic to effectively wean habitués away from their usual triggers to consume tobacco. In countries such as India, it is a public health challenge to teach SLT habitués to frequently wash their hands with soap and water or alcohol-based hand sanitizer (approved by the Centers for Disease Control and Prevention) to ensure optimal hand hygiene and reduce the risk of community transmission of COVID-19 [1].

Once a novel vaccine is found to be safe and effective against COVID-19, it would be justifiable to prioritize assistance for young SLT users who are in the preparation or action transtheoretical stage [39] of quitting. This could act as an incentive to accelerate the quitting process and arrest the spread of COVID-19 infection. It is imperative to prioritize research aimed at exploring the potential association of SLT use with COVID-19 to develop evidence-based policy options.

## Conclusions

This review reveals that SLT addiction is likely to worsen the progression and prognosis of COVID-19 infection by nicotine-induced increased expression of the ACE2 receptor and action of the furin enzyme in the oral cavity. Furthermore, the use of SLT also increases the transmission of infection by users who engage in public spitting, frequent hand-to-mouth contact, and sharing of tobacco apparatus. Hence, we cannot overlook the fact that cessation of SLT product usage by habitués can contribute to reducing the risk of infection and spread of COVID-19. At the outset, the trajectory of COVID-19 worldwide is in the hands of citizens and those who can initiate and propagate a change in regional and global tobacco control policies.
